# Understanding the Duration and Challenges of Completing Long Case Psychotherapy in Core Psychiatry Training

**DOI:** 10.1192/bjo.2025.10307

**Published:** 2025-06-20

**Authors:** Shehroz Shakeel, Laura Miller, Alina Vaida

**Affiliations:** Coventry and Warwickshire Partnership Trust, Coventry, United Kingdom

## Abstract

**Aims:** Trainees in core psychiatry training are expected to complete at least 20 sessions of long case psychotherapy within 6 months. However, many take significantly longer. This study explores the typical duration of long case psychotherapy and the challenges that contribute to delays.

**Methods:** Data was collected from the psychotherapy tutor who supervised 16 trainees who had completed their long case. Additionally, a survey was conducted among 12 trainees to identify factors contributing to delays.

**Results:** Among the 16 trainees, only 4 (25%) completed their long case within 6 months. 3 completed in 7–8 months, 3 in 9–10 months, 3 in 11–12 months and 3 took 13–15 months.

Survey results showed that among 12 respondents, only 2 (16.6%) completed their long case within 6 months, 3 took 8 months, 2 took 9 months, 2 took 10 months, and then 3 required more than 14 months.

Session cancellations emerged as a significant factor in delays. Regarding patient non-attendance, 6 trainees reported 1–3 missed sessions, 2 reported 4–6 missed sessions, 1 reported 7–9 missed sessions, and 2 reported over 10 missed sessions. Trainees themselves cancelled 1–3 sessions (7 trainees), 4–6 sessions (2 trainees), and 7–9 sessions (1 trainee). The reasons for trainee cancellations included out-of-hours commitments (60%), annual leave (80%), sick leave (30%), and study leave (60%).

The longest gap between sessions was reported as 3 weeks by 41.7% of trainees, 4 weeks by 8.3%, and more than 4 weeks by 16.7%. 66.7% of trainees believed breaks affected therapeutic relationships. Furthermore, 63.6% reported that cancellations often led to consecutive missed sessions. To maintain continuity, 75% of trainees had to conduct sessions on their scheduled days off.

However, 72.7% of trainees were satisfied with the therapy they provided, although only 54.5% believed their patients were satisfied with the treatment received.

**Conclusion:** The expected 6-month completion for long case psychotherapy is rarely achieved, with most trainees requiring longer due to cancellations, scheduling conflicts, and patient non-attendance. Long breaks between sessions negatively impact rapport, and many trainees resort to working on days off to meet requirements. Learning from colleagues that the long case takes considerably longer than initially anticipated, we aim to change the psychotherapy planning to ensure that all psychiatry residents start their long case as early as possible, ideally during CT2, with communication regarding the importance of consistent attendance.

